# Outcomes of Operative Versus Nonoperative Management for Hallux Rigidus: A Tertiary Care Center Experience

**DOI:** 10.7759/cureus.46991

**Published:** 2023-10-13

**Authors:** Ali S Alshehri, Faisal A Alzahrani, Lujain S Alqahtani, Khalid H Alhadlaq, Halah A Alshabraqi, Ziad A Aljaafri

**Affiliations:** 1 Orthopedic Surgery, King Abdulaziz Medical City, Ministry of National Guard – Health Affairs, Riyadh, SAU; 2 Orthopedic Surgery, King Abdullah International Medical Research Center, Riyadh, SAU; 3 College of Medicine, King Saud Bin Abdulaziz University for Health Sciences, Riyadh, SAU

**Keywords:** operative fixation, hallux disorders, complications, managements, hallux rigidus

## Abstract

Background

This study aims to investigate and report the outcomes of various management modalities used for hallux rigidus, a common form of degenerative joint disease affecting the foot and ankle. The research focuses on understanding the pathophysiology, classification systems, and nonoperative approaches such as medical therapy, intra-articular injections, shoe modifications, and physical therapy. Surgical techniques, including joint-sparing and joint-sacrificing procedures, are explored, considering factors such as disease stage and patient preferences.

Methods

A retrospective cohort study was conducted at King Abdulaziz Medical City (KAMC), Riyadh. The study included all patients who were diagnosed with hallux rigidus from the period 2016 to 2022. Data were collected through the BESTCare system at KAMC. All the data were collected through Microsoft Excel (Microsoft Corporation, Redmond, Washington) and transferred for analysis. Statistical analysis was performed using the IBM SPSS Statistics for Windows, Version 25 (Released 2017; IBM Corp., Armonk, New York). Frequencies and percentages were used to detail categorical variables, whereas continuous variables were examined by the mean and standard deviation. A p-value of <0.05 was considered to report the statistical significance.

Results

A total of 84 patients were included. The majority were women (60.7%). Diabetes and hypertension were prevalent comorbidities, affecting 21.4% and 35.7% of patients, respectively. Nonoperative management was the most common approach (66.7%). Complications were minimal (2.4% infections, 1.2% metatarsalgia), and 67.9% of patients reported no persistence of symptoms after treatment.

Conclusion

The low complication rates and the lack of significant associations between treatment modalities and outcomes suggest the generally safe and effective nature of the employed interventions. These findings can guide clinicians in making informed decisions regarding the management of hallux rigidus, while also highlighting areas for further research to improve treatment strategies and outcomes.

## Introduction

Hallux rigidus is a degenerative joint disease of the foot, and it is the final stage of arthritis of the first metatarsophalangeal joint [1]. It is the most prevalent type of foot arthritis, affecting 1 in 40 people over 50, with a 2:1 female prevalence [2,3]. Regarding pathophysiology it is most often idiopathic, and despite its idiopathic nature, multiple studies identified an association between the development of hallux rigidus and the damage of articular cartilage via traumatic and nontraumatic causes [4,5]. Moreover, it is characterized by osteophyte formation, narrowing joint space, decreased motion, and pain. Consequently, people with hallux rigidus experience decreased sagittal plane range of motion. Patient activity and quality of life suffer significantly as a result of this change in gait mechanics [6,7].

The two most popular classification systems are the Regnauld classification system, which categorize hallux rigidus in three grades based on the clinical presentations of the patients ranging from mild limitations in dorsiflexion in the first grade to the boarding and flattening of the metatarsal head and proximal phalangeal base, in the second grade, and finally in the third grade there is the loss of joint space and near ankylosis in addition to extensive osteophytes formation [8]. The second classification used is Coughlin and Shurnas classification system and considered to be more comprehensive, it categorizes the patients into five grades starting from zero up to four, and has three main components that include dorsiflexion, radiographic findings, and clinical findings as well [4].

In regard to the management of hallux rigidus, hallux rigidus should be treated nonoperatively before undergoing surgical procedures. Some of these methods include medical therapy, intra-articular injections, shoe modifications, activity modifications, and physical therapy. Oral nonsteroidal anti-inflammatory drugs are the mainstay of medical treatment, and they are used for joint pain and swelling. However, oral medications alone are not enough and should be combined with other modalities of nonoperative management [9]. Moreover, intra-articular injections have shown to be effective in some patients with hallux rigidus. A study showed that the benefits of intra-articular injections may last up to six months in patients with early stages of the disease and the benefit time gets shorter with the disease progression and patients may ultimately need surgical intervention [10].

Surgical modalities should be attempted for the management of hallux rigidus once nonoperative modalities fail. Surgical techniques can be grouped into two categories: the first is joint-sparing and the other is joint sacrificing. These techniques include cheilectomy which is a joint-sparing technique and is considered to be the treatment of choice for the early stages of the disease, it improves dorsiflexion and gait and reduces pain as well, with great success rate and few complications [11]. Other techniques can be tried including cheilectomy with Moberg osteotomy which can have better outcomes in moderate stages of the disease with the improvement of dorsiflexion [12]. However, for severe, end-stage disease, arthrodesis of the first MTP joint is considered and accepted to be the standard of care due to its perceived safety and efficacy [9,13-17].

Finally, to our knowledge, there are limited studies conducted in the region about the outcomes of the modalities used for the management of hallux rigidus. Therefore, we would like to study and report the outcomes of cases managed at our institution, King Abdulaziz Medical City, Riyadh.

## Materials and methods

This retrospective cohort study was conducted at King Abdulaziz Medical City (KAMC), a tertiary care center in Riyadh. The sample size was determined by including all the patients who were diagnosed with hallux rigidus and were treated either operatively or nonoperatively from the period 2016 to 2022. No exclusion criteria were implemented. 

Data were collected through the BESTCare system at KAMC. We used a data collection sheet that was prepared by the research team based on data of interest. The data collection sheet included demographics, comorbidities, nonoperative management if initiated and its modality, duration of nonoperative management, type of operative management, persistence of pain following treatment, and complications if reported. 

All the data were collected through Microsoft Excel (Microsoft Corporation, Redmond, Washington, USA) and transferred for analysis. Data were checked for any missing information, and new variables were recorded and computed based on the data extracted. Statistical analysis was performed using the IBM SPSS Statistics for Windows, Version 25 (Released 2017; IBM Corp., Armonk, New York) software. Frequencies and percentages were used to detail categorical variables, whereas continuous variables were examined by the mean and standard deviation. A p-value <0.05 was considered to report the statistical significance.

Ethical approval was obtained from King Abdullah International Medical Research Center (KAIMRC). Consent was not required for this retrospective cohort study, and all data were kept safe. No identification data were asked, and privacy and confidentiality were assured. Access to research data was kept only between the study group members.

## Results

Table 1 shows the sociodemographic and other characteristics of hallux rigidus patients. A total of 84 patients were included in the study. In terms of gender, 39.3% were men (33 patients), while the majority, 60.7%, were women (51 patients). When considering age, 6.0% of the patients were younger than 40 years (5 patients), 58.3% were between 41 and 60 years (49 patients), and 35.7% were over 60 years (30 patients). Regarding the body mass index (BMI), 10.7% had a normal BMI (9 patients), 35.7% were classified as overweight (30 patients), 31.0% fell into the category of obese class 1 (26 patients), 20.2% were classified as obese class 2 (17 patients), and only 1.2% fell into the obese class 3 category (1 patient). Regarding HbA1c, 10.7% of the patients had normal HbA1c levels (9 patients), 11.9% had pre-diabetes (10 patients), and 21.4% had diabetes (18 patients). Regarding the diagnosis age of hallux rigidus, 11.9% were diagnosed before the age of 40 (10 patients), 69.0% were diagnosed between the ages of 41 and 60 (58 patients), and 19.0% were diagnosed after the age of 60 (16 patients).

**Table 1:** The Sociodemographic and other features of hallux rigidus patients BMI: body mass index, HbA1c: hemoglobin A1c.

		Frequency (n=84)	Percentage
Gender	Men	33	39.3
	Women	51	60.7
Age (years)	<40 years	5	6.0
	41-60 years	49	58.3
	>60 years	30	35.7
BMI	Normal	9	10.7
	Overweight	30	35.7
	Obese Class 1	26	31.0
	Obese Class 2	17	20.2
	Obese Class 3	1	1.2
HbA1c	Normal	9	10.7
	Pre-diabetes	10	11.9
	Diabetes	18	21.4
Age at diagnosis of hallux rigidus	<40 years	10	11.9
	41-60 years	58	69.0
	>60 years	16	19.0

**Figure 1:**
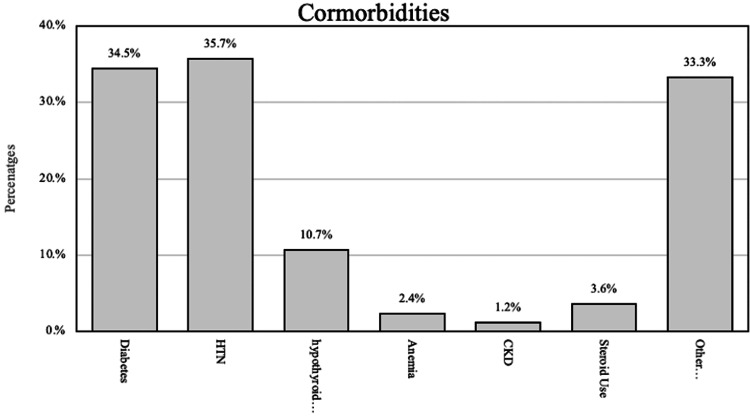
Different comorbidities in hallux rigidus patients HTN: hypertension, CKD: chronic kidney disease.

Various comorbidities among hallux rigidus patients are shown in Figure 1. In all, 34.5% of the patients had diabetes, indicating a relatively high prevalence of this condition among individuals with hallux rigidus. Hypertension (HTN) was reported in 35.7% of the patients, indicating a significant proportion of individuals with coexisting HTN. Hypothyroidism was found in 10.7% of the patients, while anemia and chronic kidney disease (CKD) were less common, affecting 2.4% and 1.2% of the patients, respectively.

Table 2 shows the features of hallux rigidus, including the rate and types of operative and nonoperative management, complications, and outcomes. All 84 patients in the study were diagnosed with hallux rigidus. Among the cases, 73.8% had the condition on one side (unilateral), while 26.2% had it on both sides (bilateral).

**Table 2:** Features of hallux rigidus with complication and outcome MO: Morton’s orthosis, N/A: not applicable.

		Frequency (n=84)	Percentage
Diagnosis	Hallux rigidus	84	100.0
Laterality	Unilateral	62	73.8
	Bilateral	22	26.2
Management modalities	Operative	17	20.2
	Nonoperative	56	66.7
	Both	11	13.1
Type of nonoperative management	Analgesia	9	10.7
	Morton’s orthosis	49	58.3
	MO and injection	9	10.7
	N/A	17	20.2
Type of operative management	Cheilectomy	16	19.0
	Fusion	11	13.1
	N/A	56	66.7
Complications	No	82	97.6
	Infection	1	1.2
	Metatarsalgia	1	1.2
Persistence of symptoms and outcome	No	57	67.9
	Yes	17	20.2
	In management	2	2.4
	No F/U	8	9.5

In terms of management modalities, 20.2% of the patients underwent operative procedures, 66.7% received nonoperative management, and 13.1% received a combination of both. Among the nonoperative management options, analgesia was used in 10.7% of the cases, Morton's orthosis (a type of foot support) was utilized in 58.3% of the cases, and 10.7% received Morton's orthosis in combination with injections. Additionally, 20.2% of the patients did not have a specific nonoperative management type mentioned (N/A).

For the operative management of hallux rigidus, cheilectomy (surgical removal of bone spurs) was performed in 19.0% of the cases, fusion (arthrodesis) was carried out in 13.1% of the cases, and 66.7% did not undergo any operative management (N/A).

Regarding complications, the majority of patients (97.6%) did not experience any complications. However, 1.2% of the patients had infections, and another 1.2% had metatarsalgia (pain in the ball of the foot).

In terms of persistence of symptoms and outcome, 67.9% of the patients reported no persistence of symptoms after management, indicating successful treatment. However, 20.2% reported that their symptoms persisted, 2.4% were still in the management phase, and 9.5% did not have any follow-up information available.

**Figure 2:**
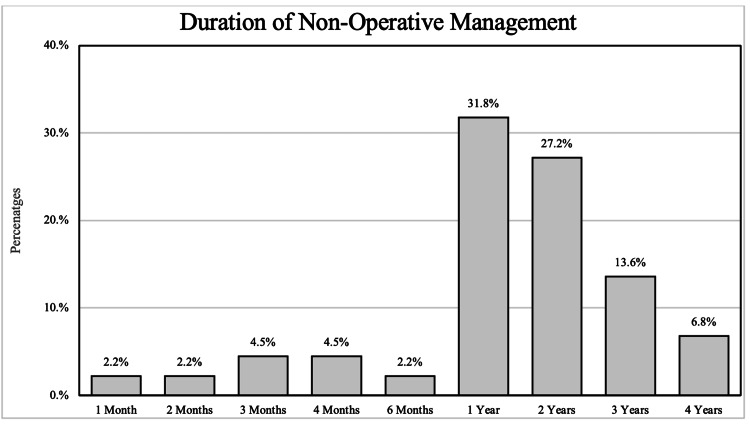
Percentages of duration of nonoperative management

Figure 2 shows the duration of nonoperative management among hallux rigidus patients. According to the data, 2.2% of patients underwent nonoperative management for one month, as well as for two months and six months each. Meanwhile, 4.5% of patients received nonoperative management for three months and four months, respectively.

The majority of patients, 31.8%, underwent nonoperative management for one year. A significant portion, 27.2%, received nonoperative treatment for two years. After that, the duration of nonoperative management gradually decreased, with 13.6% of patients receiving treatment for three years, 6.8% for four years, and so on.

Table 3 shows the association of outcomes with operative and nonoperative management, as well as other features, in hallux rigidus patients. Regarding the modality of management, there is no significant association between operative or nonoperative management and the persistence of symptoms or being in the management phase. Similarly, the type of nonoperative management (analgesia, Morton's orthosis, and Morton's orthosis and Injection) does not show a significant association with outcomes. The type of operative management, specifically cheilectomy or fusion, also does not demonstrate a significant association with outcomes. Analyzing the sociodemographic features, there are no significant associations between gender, age, BMI categories, HbA1c levels, or age at diagnosis with the persistence of symptoms or being in the management phase. Examining comorbidities, the presence of diabetes (DM), HTN, hypothyroidism, CKD, or steroid use does not show significant associations with outcomes. However, the presence of other comorbidities does demonstrate a borderline significant association with outcomes.

**Table 3:** Association of outcomes with operative and nonoperative management HbA1c: Hemoglobin A1c.

		Persistence of Symptoms			Sig. Value
		No	Yes	In Management	
Modality of management	Operative	14(24.6)	3(17.6)	0(0)	0.180
	Nonoperative	32(56.1)	14(82.4)	2(100)	
	Both	11(19.3)	0(0)	0(0)	
Type of nonoperative management	Analgesia	5(11.6)	1(7.1)	0(0)	0.859
	Morton's orthosis	32(74.4)	10(71.4)	2(100)	
	Morton's orthosis and Injection	6(14)	3(21.4)	0(0)	
Type of operative management	Cheilectomy	13(54.2)	3(100)	0(0)	0.128
	Fusion	11(45.8)	0(0)	0(0)	
Sociodemographic features					
Gender	Men	24(42.1)	4(23.5)	1(50)	0.361
	Women	33(57.9)	13(76.5)	1(50)	
Age	<40 years	5(8.8)	0(0)	0(0)	0.415
	41-60 years	30(52.6)	13(76.5)	1(50)	
	>60 years	22(38.6)	4(23.5)	1(50)	
Body mass index (BMI)	Normal	6(10.5)	2(12.5)	0(0)	0.928
	Overweight	19(33.35)	8(50)	1(50)	
	Obese Class 1	18(31.6)	4(25)	1(50)	
	Obese Class 2	13(22.8)	2(12.5)	0(0)	
	Obese Class 3	1(1.8)	0(0)	0(0)	
HbA1c	Normal	4(7)	4(23.5)	0(0)	0.325
	Prediabetes	7(12.3)	2(11.8)	0(0)	
	Diabetes	46(80.7)	11(64.7)	2(100)	
Age at diagnosis	<40 years	8(14)	1(5.9)	0(0)	0.676
	41-60 years	39(68.4)	13(76.5)	1(50)	
	>60 years	10(17.5)	3(17.6)	1(50)	
Comorbidities					
Diabetes mellitus (DM)	No	34(60.7)	13(76.5)	2(100)	0.284
	Yes	22(39.3)	4(23.5)	0(0)	
Hypertension (HTN)	No	37(66.1)	11(64.7)	2(100)	0.595
	Yes	19(33.9)	6(35.3)	0(0)	
Hypothyroidism	No	51(91.1)	14(82.4)	1(50)	0.15
	Yes	5(8.9)	3(17.6)	1(50)	
Chronic kidney disease (CKD)	No	55(98.2)	17(100)	2(100)	0.842
	Yes	1(1.8)	0(0)	0(0)	
Steroid use	No	55(98.2)	16(94.1)	2(100)	0.638
	Yes	1(1.8)	1(5.9)	0(0)	
Other comorbidities	No	40(71.4)	8(47.1)	2(100)	0.105
	Yes	16(28.6)	9(52.9)	0(0)	

Table 4 shows the association of complications with operative and nonoperative management, as well as other features in hallux rigidus patients. Regarding the modality of management, there is no significant association between operative or nonoperative management and the occurrence of complications. The majority of patients in both groups did not experience complications, with a small proportion of patients in the nonoperative group reporting metatarsalgia.

**Table 4:** Association of complications with operative and nonoperative management HbA1c: hemoglobin A1c.

		Complications			Sig. Value
		No	Infection	Metatarsalgia	
Modality of management	Operative	16(19.5)	1(100)	0(0)	0.346
	Nonoperative	55(67.1)	0(0)	1(100)	
	Both	11(13.4)	0(0)	0(0)	
Type of nonoperative management	Analgesia	9(13.6)	0(0)	0(0)	0.038
	Mortons orthosis	49(74.2)	0(0)	0(0)	
	MO and injection	8(12.1)	0(0)	1(100)	
Type of operative management	Cheilectomy	16(61.5)	0(0)	0(0)	0.219
	Fusion	10(38.5)	1(100)	0(0)	
Sociodemographic features					
Gender	Male	33(40.2)	0(0)	0()	0.515
	Female	49(59.8)	1(100)	1(0)	
Age	<40 years	4(4.9)	1(100)	0(0)	0.001
	41-60 years	49(59.8)	0(0)	0(0)	
	>60 years	29(35.4)	0(0)	1(100)	
Body mass index (BMI)	Normal	8(9.9)	1(100)	0(0)	0.231
	Overweight	30(37)	0(0)	0(0)	
	Obese Class 1	25(30.9)	0(0)	1(100)	
	Obese Class 2	17(21)	0(0)	0(0)	
	Obese Class 3	1(1.2)	0(0)	0(0)	
HbA1c	Normal	9(11)	0(0)	0(0)	0.963
	Pre-diabetes	10(12.2)	0(0)	0(0)	
	Diabetes	63(76.8)	1(100)	1(100)	
Age at diagnosis	<40 years	9(11)	1(100)	0(0)	0.095
	41-60 years	57(69.5)	0(0)	1(100)	
	>60 years	16(19.5)	0(0)	0(0)	
Comorbidities					
Diabetes mellitus (DM)	No	53(65.4)	1(100)	0(0)	0.301
	Yes	28(34.6)	0(0)	1(100)	
Hypertension (HTN)	No	52(64.2)	1(100)	0(0)	0.311
	Yes	29(35.8)	0(0)	1(100)	
Hypothyroidism	No	72(88.9)	1(100)	1(100)	0.883
	Yes	9(11.1)	0(0)	0(0)	
Chronic kidney disease (CKD)	No	80(98.8)	1(100)	1(100)	0.988
	Yes	1(1.2)	0(0)	0(0)	
Steroid use	No	78(96.3)	1(100)	1(100)	0.962
	Yes	3(3.7)	0(0)	0(0)	
Other comorbidities	No	53(65.4)	1(100)	1(100)	0.594
	Yes	28(34.6)	0(0)	0(0)	

Looking at the type of nonoperative management, there is a significant association with complications. Patients treated with Morton’s orthosis had a lower incidence of complications compared to those using analgesia or Morton’s orthosis and Injection.

Regarding the type of operative management, specifically cheilectomy or fusion, there is no significant association with complications. Both procedures had relatively low rates of complications.

Analyzing sociodemographic features, there were no significant associations between complications and gender, BMI categories, HbA1c levels, or age at diagnosis. However, the age groups of patients had significant differences as p < 0.001. Considering comorbidities, the presence of DM, HTN, hypothyroidism, CKD, steroid use, or other comorbidities did not show significant associations with complications. 

## Discussion

In terms of sociodemographic characteristics, our study demonstrated a higher prevalence of hallux rigidus among women (60.7%). Regarding age distribution, our study found that the majority of patients (58.3%) were between 41 and 60 years old, while 35.7% were over 60 years old. Regarding BMI distribution, 35.7% of patients were classified as overweight, 31.0% fell into the obese class 1 category, and 20.2% were classified as obese class 2. 

The prevalence of comorbidities in hallux rigidus patients is an important consideration. Our study revealed a relatively high prevalence of diabetes (21.4%) and hypertension (35.7%) among these patients. These findings align with the prior research, which also reported a higher incidence of diabetes and hypertension in individuals with hallux rigidus [18]. The presence of these comorbidities may have implications for treatment and management strategies, as well as the overall prognosis of hallux rigidus patients [19].

In terms of management modalities, our study demonstrated that a majority of patients (66.7%) received nonoperative management, while 20.2% underwent operative procedures. This distribution is consistent with the study by Kon Kam King et al., who reported a similar utilization of nonoperative and operative management options in their study [20]. Nonoperative management options, including analgesia and the use of Morton’s orthosis, were commonly employed in our study, reflecting their role in alleviating symptoms and improving functional outcomes [21].

Among the operative management options, cheilectomy (19.0%) and fusion (13.1%) were the most frequently performed procedures in our study. These findings are in line with previous studies, which reported comparable rates of cheilectomy and fusion in the surgical management of hallux rigidus [22,23]. 

Regarding complications, our study found that the majority of patients (97.6%) did not experience any complications. This suggests that both operative and nonoperative management approaches were generally safe and well tolerated in hallux rigidus patients. The low rate of complications aligns with previous research by Grimm et al., who also reported minimal complications associated with surgical interventions for hallux rigidus [24].

Our study showed that a significant proportion of patients (67.9%) reported no persistence of symptoms following management, indicating successful treatment. This finding is consistent with the research conducted by Miettinen et al., who reported favorable outcomes in terms of symptom relief and functional improvement in hallux rigidus patients treated with various modalities [25]. 

When analyzing the association between outcomes and management modalities, our study did not find any significant associations between operative or nonoperative management and the persistence of symptoms or being in the management phase. This suggests that both approaches can yield comparable outcomes in terms of symptom relief. These findings are in line with the study, which also reported similar outcomes between surgical and nonsurgical interventions for hallux rigidus [26].

The type of nonoperative management, including analgesia, Morton’s orthosis, and Morton’s orthosis and injection, did not show a significant association with outcomes in our study. This finding is consistent with previous research by Mikko et al. and Deborah et al., who also did not find significant differences in outcomes among different nonoperative treatment modalities [25,27]. It suggests that the choice of nonoperative management may depend on individual patient preferences and clinician expertise.

Similarly, the type of operative management, namely cheilectomy or fusion, did not demonstrate a significant association with outcomes in our study. These findings are supported by the research, which reported comparable outcomes between cheilectomy and fusion procedures [28]. The selection of the surgical approach may depend on the severity of the condition, joint deformities, and patient-specific factors [29].

Analyzing sociodemographic features, our study did not find significant associations between gender, age, BMI categories, HbA1c levels, or age at diagnosis with the persistence of symptoms or being in the management phase. These results are consistent with previous studies by Sánchez-Gómez et al. and Molines-Barroso et al., which also did not observe significant associations between these factors and treatment outcomes in hallux rigidus patients [18,30]. 

In terms of complications, our study did not find significant associations between operative or nonoperative management and the occurrence of complications. Patients treated with Morton’s orthosis had a lower incidence of complications compared to those using analgesia or a combination of Morton’s orthosis and injections. This finding suggests that Morton’s orthosis may offer a safer nonoperative treatment option with fewer complications. These results are in line with the study, which reported favorable outcomes and lower complication rates with the use of orthotic devices in hallux rigidus patients [31].

Regarding the association between complications and sociodemographic features, our study did not find significant associations with gender, BMI categories, HbA1c levels, or age at diagnosis. In terms of comorbidities, our study did not identify significant associations between complications and the presence of diabetes, hypertension, hypothyroidism, chronic kidney disease, or steroid use. 

Regarding the limitations of the current study, the data were gathered from one center, which might restrict the generalizability of the outcomes. In addition, this topic needs more exploration with a larger sample size and multiple center involvement.

## Conclusions

Our study provides valuable insights into the demographic characteristics, management approaches, complications, and treatment outcomes of hallux rigidus patients. The low complication rates and the lack of significant associations between treatment modalities and outcomes suggest the generally safe and effective nature of the employed interventions. These findings can guide clinicians in making informed decisions regarding the management of hallux rigidus patients, while also highlighting areas for further research to improve treatment strategies and outcomes.
